# Expanding range of ***Ixodes scapularis*** Say (Acari: Ixodidae) and *Borrelia burgdorferi* infection in North Carolina counties, 2018–2023

**DOI:** 10.1371/journal.pone.0329511

**Published:** 2025-08-13

**Authors:** Reuben A. Garshong, Dayvion R. Adams, Steven W. Seagle, Gideon Wasserberg, Michael H. Reiskind, Jimmie L. Teague, Carl J. Williams, Alexis M. Barbarin

**Affiliations:** 1 Biology Department, University of North Carolina at Greensboro, Greensboro, North Carolina, United States of America; 2 Department of Entomology and Plant Pathology, North Carolina State University, Raleigh, North Carolina, United States of America; 3 Department of Biology, Appalachian State University, Boone, North Carolina, United States of America; 4 North Carolina Department of Health and Human Services, Raleigh, North Carolina, United States of America; UNAM: Universidad Nacional Autonoma de Mexico, MEXICO

## Abstract

North Carolina (NC) has been experiencing a recent surge in human Lyme disease (LD) cases. Understanding the distribution of tick-borne diseases necessitates understanding the distribution of the ticks that transmit their causative pathogens. Unfortunately, in NC, knowledge on tick distribution is outdated. In this manuscript, we report the results of a state-wide entomologic survey conducted in 42 NC counties by flagging/dragging from spring 2018 to summer 2023. *Ixodes scapularis* nymphs and adults were screened for *Borrelia burgdorferi* (the causative agent of LD) and four other tick-borne bacterial pathogens (*Anaplasma phagocytophilum*, *B. mayonii*, *B. miyamotoi, and Babesia microti*) by the Centers for Disease Control and Prevention (CDC). Consistent with current data on human LD cases incidence and distribution, results of this study indicated a range expansion of *I. scapularis* with higher tick densities and *B. burgdorferi* infection prevalence now occurring in the Blue Ridge Mountains province of western NC. Temporal analysis of *I. scapularis* presence data indicated that this shift is fairly recent (about 10 years). Finally, in the Blue Ridge Mountains we detected a northeast-to-southwest gradient in *I. scapularis* tick and *B. burgdorferi* infection prevalence suggesting that this trend is driven by a spread of the northern clade *I. scapularis* ticks into NC from southwestern Virginia, along the Appalachian Mountains. Other pathogenic bacteria detected in *I. scapularis* ticks included *B. miyamotoi* and *A. phagocytophilum*, that were limited to the Blue Ridge Mountains*.* These results have important public health implications, including the need for enhanced tick surveillance, updated clinical awareness, and targeted public education in newly affected areas.

## Introduction

Lyme disease (LD) is the most prevalent arthropod-borne zoonotic disease in the United States (US) [1], accounting for over 70% of all reported vector-borne disease cases in the US [2]. The disease in the US is caused by the spirochete bacteria, *Borrelia burgdorferi* (Bb) *sensu stricto*, and rarely, *Borrelia mayonii.* In the eastern US, the blacklegged tick, *Ixodes scapularis* Say, transmits the pathogen to humans, and is likely responsible for almost all human LD cases [3].

Since its discovery in Connecticut, USA, in the 1970s, LD has surged in geographic extent and number of cases [4], reaching at least 62,551 human cases in 2022 [5]. Currently, 16 states mostly in the Northeast, mid-Atlantic, and upper Midwest account for 95% of the reported LD cases [5]. However, low incidence states including North Carolina (NC), have experienced an increase in the number of LD cases [5]. Specifically, using a spatiotemporal cluster analysis of human cases in Virginia, Lantos et al. [6] described a temporal change in LD clusters from the northeast towards the southwest, along the Appalachian Mountains, suggesting an imminent threat of spread of LD into NC. This work was followed by an analysis of the spatiotemporal dynamics of LD in NC from 2010 to 2020 [7]. That study found that the greatest magnitude of increase occurred in the northwestern parts of the state while in the coastal and Piedmont provinces of NC little or no change in LD incidence have occurred. Incidence within the high transmission clusters was found to be comparable to that reported in high incidence regions of the northeast and Midwest US [7]. Furthermore, that study reported that during that period the leading edge of high-incidence zip codes moved approximately 80–100 miles in a southwestward direction along the Blue Ridge Mountains.

These studies [6,7] suggested that this change in human LD cases, might be driven by an invasion of northern clade ticks spreading southwestwards along the Appalachian Mountain chain into NC resulting in an apparent westward expansion of *I. scapularis* and *B. burgdorferi* range in NC. Unfortunately, knowledge on tick distribution in NC is outdated, with historical data indicating an endemic distribution of *I. scapularis* mainly in the coastal region (rare in the Piedmont and absent in the mountains) with ticks tending to feed mostly on lizards [8–11]. These studies suggested that this tick population belongs to the southern clade which tends to quest underneath the leaf litter and is less anthropophilic compared with northern clade ticks [12,13].

The goal of this study was to re-evaluate the distribution of *I. scapularis* and Bb in NC, with a particular focus on testing the hypothesis of a southwestward invasion of *I. scapularis* along the Blue Ridge Mountains. Specifically, first, we compared nymph and adult *I. scapularis* densities and Bb infection rates among the three NC physiographic provinces (the Blue Ridge Mountains, Piedmont, and Coastal plain) and analyzed the temporal change in *I. scapularis* presence status throughout NC. Second, for the Blue Ridge Mountains province, we evaluated evidence for a north-to-south gradient in nymph and adult *I. scapularis* densities and their respective Bb infection rates. We also evaluated and compared the presence of other tick-borne bacteria such as *Anaplasma phagocytophilum*, *Babesia microti*, *Borrelia mayonii*, and *Borrelia miyamotoi* in *I. scapularis* ticks among the three physiographic provinces.

## Methods

### Study area

North Carolina’s landscape is shaped by three main physiographic provinces: the Coastal Plain, Piedmont, and Mountains. The Coastal Plain (in the eastern part of the state) is flat and low in elevation, with sandy soils, wetlands, and slow-moving rivers. It has a warm, humid climate. Vegetation includes pine forests, marsh grasses, and swamp hardwoods. Land use centers on agriculture (tobacco, cotton, soybeans), forestry, and tourism. The Piedmont (in the central part of the state) rises gradually westward with rolling hills and elevations from about 90–450 meters above sea level. The soil is mainly red clay, and the region has a moderate climate. Originally, hardwood forests covered much of the area, but a lot of it has been cleared for cities, suburbs, and farmland. It is now the economic center of the state, supporting urban development, education, and light industry. The Mountain province, located in the western part of the state and forming part of the Appalachian Mountain chain, is characterized by rugged terrain with some of the highest elevations in the eastern United States. The climate in this region is notably cooler, with higher levels of precipitation and snowfall at elevated altitudes. The landscape is dominated by dense forests composed of spruce, fir, and various hardwood species. Due to the steep and uneven terrain, large-scale agriculture is limited. Instead, land use in this region is primarily focused on tourism, forestry, and small-scale agricultural activities (see S3 Fig).

### Survey design

Tick surveillance in this study was conducted between May 2018 and July 2023 across 42 counties in NC (see S2 Fig). Our survey focused primarily on the Piedmont (19 counties) and Blue Ridge Mountains physiographic provinces (19 counties), where the most notable changes in human LD case reports have occurred [7]. In the coastal region, we sampled in four counties with prior reports of human LD cases [14]. Each year, three teams of the participating universities (University of North Carolina at Greensboro, Greensboro, North Carolina State University, and Appalachian State University) sampled counties in their respective regions. In most counties, each team sampled two sites, typically state parks or other public lands, with suitable *I. scapularis* habitat, generally characterized by deciduous or pine forest cover [15–17]. In four counties (one coastal, three Piedmont), only one site was sampled due to a lack of public lands meeting our criteria. The number of transects per site ranged from 12 to 18, depending on patch size, with all transects spaced at least 100 meters apart. In sites with particularly small forest patches, fewer transects were conducted. Tick sampling was performed biannually: once in late spring to summer (May to August) targeting nymphs, and once in fall to early spring (November to March) targeting adults. At each site, ticks were collected using 100-meter flagging/dragging transects. A 1 m² white flannel cloth attached to a 1.5 m wooden pole was dragged (at a slow pace of about 10 meters per minute) along the forest floor and inspected for attached ticks every 20–25 meters. The majority of sampled counties were surveyed for at least two years, while the remaining 10 counties were sampled for only one year. Additional data from other sampling projects in the region (e.g., [18]) specifically from Buncombe and Mecklenburg counties, were incorporated into our database. Variability in the number of sampling sites among our sampled counties was accounted for in our statistical models (see Data Analysis section). Ticks found on the cloths were collected using a pair of forceps and stored in 1.5 ml Eppendorf tubes containing 95% ethanol, one tube per transect. Each collection transect was georeferenced. The ticks were temporarily stored in the lab at −20°C. We identified ticks to species level morphologically [19,20]. Molecular confirmation of *I. scapularis* was done at the CDC.

### Pathogen screening

*Ixodes scapularis* were screened for *Anaplasma phagocytophilum*, *Babesia microti*, *Borrelia burgdorferi, Borrelia mayonii*, and *Borrelia miyamotoi*, targeting the 16s rRNA gene. Total tick DNA was extracted followed by multiplex PCR analysis at the Division of Vector-borne Diseases, CDC, Fort Collins, CO, USA, using established protocols [21–24]. Briefly, DNA was extracted using 545 mg yttria-stabilized zirconium oxide beads in Qiagen lysis buffer, disrupted via bead-beating for 2 minutes, and incubated at 56ºC for 10–12 minutes [21–22]. Samples were processed using the Qiagen automated nucleic acid isolation system and the Qiagen-Cador Pathogen 96 kit [22]. After washing and elution, DNA was screened for *Borrelia* species, *Anaplasma phagocytophilum*, and *Babesia microti* using multiplex real-time PCR assays. [23]. Modifications included a pan-*Borrelia* 16S target instead of the *B. burgdorferi* “gB31” target [24]. Positive *Borrelia* samples underwent further testing to differentiate *B. miyamotoi*, *B. burgdorferi* s.s., and *B. mayonii* via a duplex real-time PCR assay targeting the oppA2 gene [25].

### Data reduction and analysis

Tick density per county was measured by averaging the number of ticks per 100 m transect across sampling sites and sampling years for a given county (Table 1 and 2). The prevalence of *B. burgdorferi* in ticks was calculated at the county level by lumping all *I. scapularis* of a particular life stage collected over the duration of the survey years and calculating the fraction of infected ticks over the total number of ticks tested for that life stage (Table 1, 2).

**Table 1:** Summary of county-level *I. scapularis* nymphs’ abundance and infection prevalence of selected pathogenic bacteria.

Region	County	# Collected	Nymph density (s.e)	# Tested	Infection prevalence		
					Bb (s.e.)	Bm (s.e.)	Ap (s.e.)
**Blue Ridge**	Alleghany	659	5.38 (2.57)	330	0.29 (0.00)	0.00 (0.00)	0.01 (0.00)
	Ashe	136	2.12 (0.67)	121	0.39 (0.01)	0.03 (0.00)	0.01 (0.00)
	Avery	5	0.08 (0.06)	5	0.00 (0.00)	0.00 (0.00)	0.00 (0.00)
	Buncombe	295	0.80 (0.21)	143	0.13 (0.00)	0.03 (0.00)	0.01 (0.00)
	Burke	0	0.00 (0.00)	0	–	–	–
	Caldwell	11	0.21 (0.14)	11	0.00 (0.00)	0.00 (0.00)	0.00 (0.00)
	Haywood	3	0.13 (0.04)	3	0.00 (0.00)	0.00 (0.00)	0.00 (0.00)
	Henderson	2	0.04 (0.03)	2	0.00 (0.00)	0.00 (0.00)	0.00 (0.00)
	Jackson	1	0.02 (0.02)	0	–	–	–
	Macon	0	0.00 (0.00)	0	–	–	–
	Madison	24	2.00 (0.5)	24	0.08 (0.01)	0.00 (0.00)	0.00 (0.00)
	McDowell	2	0.04 (0.04)	2	0.00 (0.00)	0.00 (0.00)	0.00 (0.00)
	Mitchell	66	2.75 (0.55)	66	0.00 (0.00)	0.02 (0.00)	0.02 (0.00)
	Polk	0	0.00 (0.00)	0	–	–	–
	Rutherford	1	0.02 (0.02)	1	0.00 (0.00)	0.00 (0.00)	0.00 (0.00)
	Transylvania	0	0.00 (0.00)	0	–	–	–
	Watauga	477	3.17 (0.66)	375	0.21 (0.00)	0.00 (0.00)	0.03 (0.00)
	Wilkes	13	0.21 (0.07)	13	0.23 (0.04)	0.00 (0.00)	0.00 (0.00)
	Yancey	46	3.83 (1.5)	45	0.00 (0.00)	0.00 (0.00)	0.00 (0.00)
**Piedmont**	Alamance	0	0.00 (0.00)	0	–	–	–
	Alexander	0	0.00 (0.00)	0	–	–	–
	Anson	0	0.00 (0.00)	0	–	–	–
	Catawba	0	0.00 (0.00)	0	–	–	–
	Chatham	26	0.05 (0.01)	20	0.00 (0.00)	0.00 (0.00)	0.00 (0.00)
	Davie	0	0.00 (0.00)	0	–	–	–
	Durham	0	0.00 (0.00)	0	–	–	–
	Forsyth	0	0.00 (0.00)	0	–	–	–
	Guilford	12	0.50 (0.08)	11	0.00 (0.00)	0.00 (0.00)	0.00 (0.00)
	Iredell	0	0.00 (0.00)	0	–	–	–
	Mecklenburg	3	0.01 (0.00)	1	0.00 (0.00)	0.00 (0.00)	0.00 (0.00)
	Montgomery	0	0.00 (0.00)	0	–	–	–
	Orange	1	0.01 (0.01)	1	0.00 (0.00)	0.00 (0.00)	0.00 (0.00)
	Stanly	0	0.00 (0.00)	0	–	–	–
	Surry	46	1.10 (0.53)	32	0.03 (0.00)	0.00 (0.00)	0.00 (0.00)
	Union	0	0.00 (0.00)	0	–	–	–
	Vance	11	0.06 (0.03)	11	0.00 (0.00)	0.00 (0.00)	0.00 (0.00)
	Wake	1	0.01 (0.01)	1	0.00 (0.00)	0.00 (0.00)	0.00 (0.00)
	Yadkin	2	0.07 (0.01)	1	0.00 (0.00)	0.00 (0.00)	0.00 (0.00)
**Coastal Plain**	Craven	0	0.00 (0.00)	0	–	–	–
	Camden	3	0.13 (0.13)	3	0.00 (0.00)	0.00 (0.00)	0.00 (0.00)
	Johnston	0	0.00 (0.00)	0	–	–	–
	Onslow	0	0.00 (0.00)	0	–	–	–

Nymph density indicates average (s.e.) number of nymphs per 100 m^2^ transect. Infection prevalence (s.e.) for tested nymphs is reported for *Borrelia burgdorferi* (Bb), *B. miyamotoi* (Bm) and *Anaplasma phagocytophilum* (Ap).

**Table 2:** Summary of county level *I. scapularis* adults’ abundance and infection prevalence of selected pathogenic bacteria.

Region	County	# collected	Adult density (s.e.)	# ticks tested	Infection prevalence		
					Bb (s.e.)	Bm (s.e.)	Ap (s.e.)
**Blue Ridge Mountains**	Alleghany	96	1.33 (0.67)	47	0.62 (0.02)	0.02 (0.00)	0.00 (0.00)
	Ashe	143	1.43 (0.45)	64	0.61 (0.01)	0.00 (0.00)	0.02 (0.00)
	Avery	0	0.00 (0.00)	0	–	–	–
	Buncombe	23	0.22 (0.07)	20	0.25 (0.03)	0.05 (0.01)	0.05 (0.01)
	Burke	0	0.00 (0.00)	0	–	–	–
	Caldwell	1	0.04 (0.04)	1	0.00 (0.00)	0.00 (0.00)	0.00 (0.00)
	Haywood	10	0.42 (0.28)	10	0.00 (0.00)	0.00 (0.00)	0.00 (0.00)
	Henderson	0	0.00 (0.00)	0	–	–	–
	Jackson	0	0.00 (0.00)	0	–	–	–
	Macon	0	0.00 (0.00)	0	–	–	–
	Madison	3	0.25 (0.08)	3	0.00 (0.00)	0.00 (0.00)	0.00 (0.00)
	McDowell	0	0.00 (0.00)	0	–	–	–
	Mitchell	5	0.41 (0.19)	5	0.00 (0.00)	0.00 (0.00)	0.20 (0.09)
	Polk	0	0.00 (0.00)	0	–	–	–
	Rutherford	0	0.00 (0.00)	0	–	–	–
	Watauga	18	0.31 (0.09)	18	0.61 (0.04)	0.00 (0.00)	0.00 (0.00)
	Wilkes	6	0.17 (0.08)	6	0.83 (0.15)	0.00 (0.00)	0.00 (0.00)
	Yancey	1	0.08 (0.08)	1	0.00 (0.00)	0.00 (0.00)	0.00 (0.00)
**Piedmont**	Alamance	0	0.00 (0.00)	0	–	–	–
	Alexander	1	0.04 (0.04)	1	0.00 (0.00)	0.00 (0.00)	0.00 (0.00)
	Anson	1	0.08 (0.08)	1	0.00 (0.00)	0.00 (0.00)	0.00 (0.00)
	Catawba	0	0.00 (0.00)	0	–	–	–
	Chatham	17	0.03 (0.01)	5	0.00 (0.00)	0.00 (0.00)	0.00 (0.00)
	Davie	0	0.00 (0.00)	0	–	–	–
	Durham	0	0.00 (0.00)	0	–	–	–
	Forsyth	0	0.00 (0.00)	0	–	–	–
	Guilford	0	0.00 (0.00)	0	–	–	–
	Iredell	0	0.00 (0.00)	0	–	–	–
	Mecklenburg	0	0.00 (0.00)	0	–	–	–
	Montgomery	0	0.00 (0.00)	0	–	–	–
	Orange	1	0.003 (0.00)	1	0.00 (0.00)	0.00 (0.00)	0.00 (0.00)
	Stanly	0	0.00 (0.00)	0	–	–	–
	Surry	0	0.00 (0.00)	0	–	–	–
	Union	0	0.00 (0.00)	0	–	–	–
	Vance	5	0.03 (0.01)	5	0.00 (0.00)	0.00 (0.00)	0.00 (0.00)
	Yadkin	0	0.00 (0.00)	0	–	–	–
**Coastal Plain**	Camden	30	1.25 (1.17)	30	0.00 (0.00)	0.00 (0.00)	0.00 (0.00)
	Craven	7	0.58 (0.15)	7	0.00 (0.00)	0.00 (0.00)	0.00 (0.00)
	Johnston	4	0.16 (0.07)	3	0.67 (0.27)	0.00 (0.00)	0.00 (0.00)
	Onslow	5	0.25 (0.15)	5	0.00 (0.00)	0.00 (0.00)	0.00 (0.00)

Adult density indicates average (s.e.) number of adults per 100 m^2^ transect. Infection prevalence (s.e.) for tested adults are reported for *Borrelia burgdorferi* (Bb), *B. miyamotoi* (Bm) and *Anaplasma phagocytophilum* (Ap)

### Tick density and infection data

Given the hierarchical nature of our data (sites nested within county) we used the generalized linear mixed modeling approach (GLMM). Specifically, for the tick abundance (count data) we used Negative Binomial GLMM with ‘year’, ‘county’, and ‘site’ nested within ‘county’ as random variables. ‘Sampling effort’ was used as an offset to account for differences in sampling effort. To compare tick abundance among regions, we used our statewide data with ‘physiographic provinces’ as our predictor variable. We used a logistic GLMM approach using binomial distribution (and logit link function) to analyze Bb infection prevalence data. For testing the ‘Virginia invasion’ hypothesis, we used only data from the Blue Ridge Mountains province using a similar modeling approach for tick density and infection prevalence, but with ‘Latitude’ of sampling sites as our predictor variable. Latitude was standardized (latitude scaled) by z-transformation (mean-centered and scaled by one standard deviation) prior to analysis. Sensitivity analysis for model variables was done by comparing AIC values for different competing models. Detailed statistical tables of our GLMM analyses are provided in S4 File.

### Temporal change in *Ixodes scapularis* presence status

To evaluate the change is *I. scapularis* presence status, we used Dennis et al. [26] classification method. Specifically, counties were classified as either “established” (when at least two different life stages or six individuals of the same life stage were collected in one year), “reported” (when less than six individuals of one life stage were collected in one year), or “not detected” (when no ticks were collected). The 1996 (from 1907–1996) and 2015 (from 1907–2015) status maps were cumulative and adopted from both Dennis et al. [26] and Eisen et al. [27]. The 2023 status map was also cumulative combining our data with all past data (1907–2023).

Spatial data were visualized using quantum geographic information system (QGIS, version 3.34.6).

### Ethics

Tick samples were collected from public sites, with administrative approvals from managers of the parks and wildlife areas as necessary.

## Results

### Regional effects on tick distribution, density, and Bb infection

#### Nymphs.

**Fig 1:**
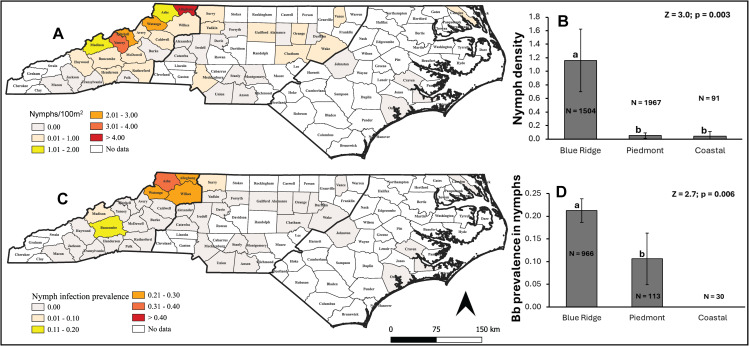
Statewide distribution and regional comparison of *Ixodes scapularis* nymph density and *Borrelia burgdorferi* infection prevalence in North Carolina. **(A)** County-level distribution of *I. scapularis* nymph densities (number per 100 m transect). Bold lines separate the three major physiogeographic provinces of North Carolina: Mountains, Piedmont, and Coastal Plain (going from west to east, respectively). **(B)** Comparison of mean nymph density (±95% CI) across the three major physiogeographic provinces of North Carolina. *N* represents the total number of transects surveyed. Z and *P* values are from a negative binomial GLMM. **(C)** County-level distribution of *B. burgdorferi* infection prevalence in *I. scapularis* nymphs (for counties with > 6 nymphs) **(D)** Comparison of *B. burgdorferi* infection prevalence (±95% binomial CI) in nymphs across the three physiogeographic provinces. *N* denotes the number of nymphs tested. Z and *P* values are derived from a weighted logistic regression. Different letters indicate statistically significant differences among regions.

**Fig 2:**
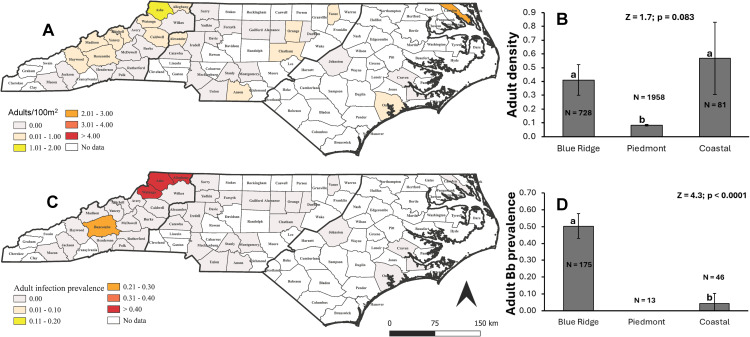
Statewide distribution and regional comparison of *Ixodes scapularis* adult tick density and *Borrelia burgdorferi* infection prevalence in North Carolina. **(A)** County-level distribution of *I. scapularis* adult densities (number per 100 m transect). Bold lines separate the three major physiogeographic provinces of North Carolina: Mountains, Piedmont, and Coastal Plain (going from from west to east, respectively). **(B)** Comparison of mean adult tick density (±95% CI) across the three major physiogeographic provinces of North Carolina. *N* represents the total number of transects surveyed. Z and *P* values are from a negative binomial GLMM. **(C)** County-level distribution of *B. burgdorferi* infection prevalence in *I. scapularis* adults (for counties with > 6 adult ticks). **(D)** Comparison of *B. burgdorferi* infection prevalence (±95% binomial CI) in adults across the three physiogeographic provinces. *N* denotes the number of nymphs tested. Z and *P* values are derived from a weighted logistic regression. Different letters indicate statistically significant differences among regions.

A total of 1,846 *I. scapularis* nymphs were collected in this study. *Ixodes scapularis* nymphs were found in all three physiographic provinces of the state (Fig 1A). However, the percentage of sampled counties with nymphs tended to be higher in the Blue Ridge Mountains (73%) (n = 19), compared to the Piedmont (42%) (n = 19), and the Coastal Plain (25%) (n = 4) (Fig 2A; χ^2^ = 5.41, p = 0.067). Consistently, nymph density was highest in the Blue Ridge Mountains province (1.16 nymphs per 100-m transect) followed by the Piedmont (0.05 nymphs per 100-m transect) and the Coastal province (0.04 nymphs per 100-m transect) (Fig 1B). *Borrelia burgdorferi* infected nymphs were found predominantly in the Blue Ridge Mountains province (Fig 1C) with 43% of counties reporting nymphs also reporting Bb infection, especially in the northern counties (Fig 1A, 1C). In contrast, the Piedmont had only 13% and the coast 0% (χ^2^ = 5.62, p = 0.060). Infection prevalence in nymphs was significantly higher in the Blue Ridge Mountains (21.2%), followed by the Piedmont (10.6%), and none in the coastal region (Fig 1D).

#### Adults.

A total of 377 *I. scapularis* adults were collected in this study. *Ixodes scapularis* adults were found in all three physiographic provinces of the state (Fig 2A). However, interestingly, the highest percentage presence was registered at the coastal region (100%) but note that this is based only on only 4 counties sampled in that region. In the Blue Ridge Mountains and the Piedmont provinces, 50% (n = 18) and 28% (n = 18) of the sampled counties were positive for the presence of adults, respectively (Fig 2A; χ^2^ = 7.23, p = 0.027). Adult density did not differ significantly between the Blue Ridge Mountains and the Coastal Plain provinces but was significantly lower in the Piedmont compared with the other two (Fig 2B). Similar to Bb infection pattern in nymphs, adult infections concentrated in the Blue Ridge Mountains with five infected counties out of 18 counties with adult ticks (27.8%) compared with one out of four in the coast (25%) and zero in the Piedmont (Fig 2A, 2C; χ^2^ = 5.80, p = 0.06). Infection prevalence in adult ticks was significantly highest in the Blue Ridge Mountains (50.3%), followed by the coast (0.04%), with 0 infections in the Piedmont (Fig 2D).

### Temporal analysis of *Ixodes scapularis* presence status in North Carolina, 1907 to present

**Fig 3:**
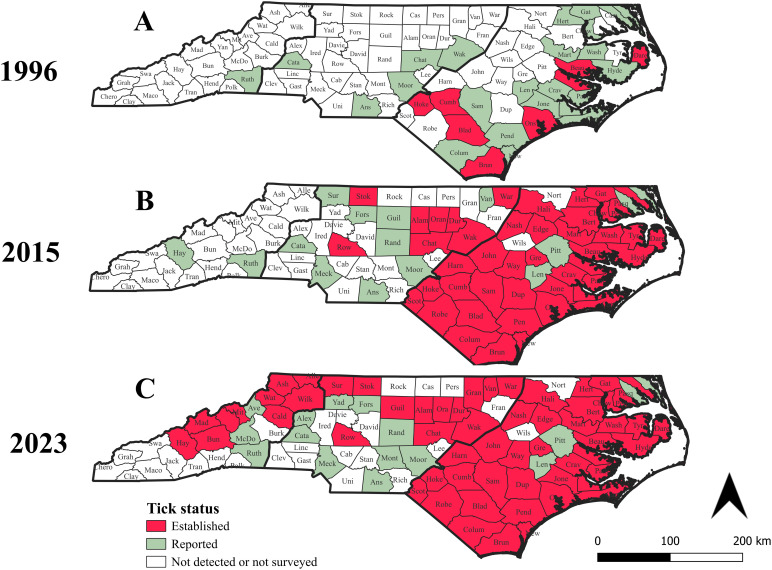
Change in presence status of blacklegged ticks (*Ixodes scapularis*) in North Carolina counties. **(A)** Presence status for 1907-1996 (Source: Dennis et al. [26]). **(B)** Cumulative presence status for 1907-2015 (Source: Eisen et al. [27]). (C.) Cumulative presence status for 1907-2023 (this study). The color of the counties indicates the ‘presence category’ status of *I. scapularis*: white for ‘not detected or not surveyed’, green for ‘reported’ (defined as < 6 individuals of a single life stage), and red for ‘established’ (defined as at least 6 individuals of the same life stage or at least a single individual of at least two different life stages collected in one year). Bold lines separate the map into the three physiographic provinces of NC: Blue Ridge Mountains (left or west), Piedmont (middle or central), and Coastal Plain (right or east).

Based on historical data and our current survey, we described the change in *I. scapularis* tick presence status from 1907 to present. In 1907–1996, counties documenting ‘reported’ or ‘established’ status occurred mostly in the Coastal Plain (Fig 3A). By 2015, all previous ‘reported’ counties have converted to ‘established’ with many new counties at each status being added, with a notable increase in the Piedmont (Fig 3B). Up until 2015, Rutherford and Haywood were the only counties in the Blue Ridge Mountains that recorded the presence of *I. scapularis* (Fig 3A, 3B). In contrast, our 2018–2023 survey introduced two novel observations. First, our study reports an extended presence of *I. scapularis* in counties within the Blue Ridge Mountains. Specifically, 13 counties reported a change in the presence status of *I. scapularis* ticks (Fig 3C). Among these, two were classified as ‘reported’ (adding on to the unchanged status in Rutherford County) and the rest as ‘established’ (Fig 3C). Second, in the Piedmont, most change in *I. scapularis* status has occurred in the western sections of this region. Specifically, three new counties containing *I. scapularis* ticks were found, two of them bordering the mountains region (Yadkin and Alexander). Another two that converted from ‘reported’ to ‘established’ (Guilford and Surry) also occurred in the western portion of the Piedmont (table related to this information is provided in S1 Table).

In summary, whereas tick presence in the Coastal Plain and Piedmont provinces changed little between 2015 and 2023, a dramatic change in *I. scapularis* distribution appears to have occurred in the Blue Ridge Mountains province.

### Tick distribution, density, and infection patterns within the Blue Ridge Mountains

Of the 19 counties sampled in the Blue Ridge Mountains province 73.7% were found positive for *I. scapularis* ticks and among these 42.9% were also found positive for Bb infection. We tested for a north-to-south gradient in tick density and Bb infection prevalence using GLMMs (see ‘data analysis’ section and Appendix) to evaluate support for the “Virginia invasion” hypothesis.

#### Nymphs.

**Fig 4:**
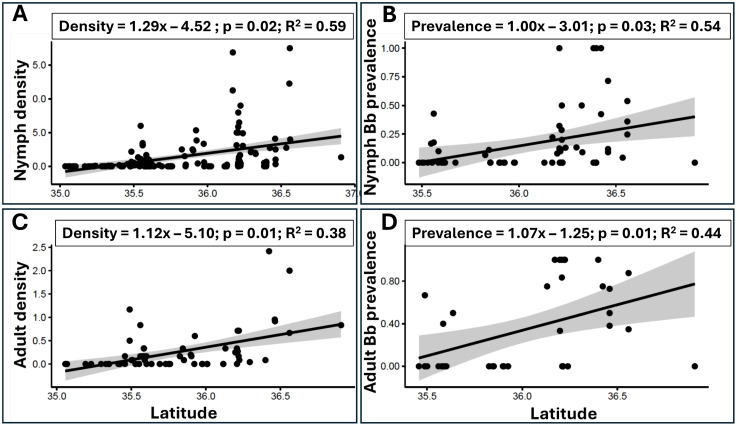
The effect of latitude (of sampling site) on *I. scapularis* density and *Bb* infection prevalence. Panels A and B, depict these patterns for nymphs and panels C and D, present these data for adult ticks. Regression equation, P-value, and R^2^ are derived from respective GLMM models (negative binomial for tick density and logistic regression for prevalence). The bands around the lines-of-best-fit are standard error margins.

A significant latitudinal effect on nymph density was found (Z = 2.36, p = 0.02) with nymph density increasing from south to north (Fig 4A). Specifically, nymphs were detected throughout most of the northern and central counties except for Avery, Burke, and McDowell counties (Fig 1A). However, among the six southernmost counties, *I. scapularis* was detected only in two: Henderson and Rutherford (Fig 1A). Highest nymph density was found in Ashe, Alleghany, and Watauga counties adjacent to the NC-Virginia border (Fig 1A). In these counties, that were also sampled continuously between 2018–2021, no significant inter-annual trend in nymph density was observed (Z = −0.40, p = 0.69). With respect to nymphs Bb infection*,* a significant (Z = 2.48, p = 0.01) effect of ‘latitude’ was found with nymphs Bb infection also increasing from south to north (Fig 4B). Infection concentrated in the four northmost counties with an additional infected area occurring in the more central Buncombe and Madison counties (Fig 1C).

#### Adults.

A significant effect of ‘latitude’ (Z = 2.18, p = 0.03) was found also for adults, with density increasing from south to north (Fig 4C). As with nymphs, adults were detected throughout most of the northern and central counties. However, among the six southernmost counties, *I. scapularis* was detected in none (Fig 2A). The highest adult density was found in Ashe County followed by Alleghany County along the NC-Virginia border (Fig 2A, Table 2). As with nymphs, a significant (Z = 2.45, p = 0.01) effect of ‘latitude’ was found with adult Bb infection increasing from south to north (Fig 4D). Infection concentrated in the three northmost counties with additional infections occurring in Buncombe County (Fig 2C).

### Infection patterns of other potential pathogens in *I. scapularis*

No infections with *Ba. microti* or *B. mayonii* were detected in the screened *I. scapularis* ticks. With *B. miyamotoi* and *A. phagocytophilum*, infection was limited to the Blue Ridge Mountains (Table 1, 2). *Borrelia miyamotoi* infection was characterized by low infection prevalence (0.03) in nymphs collected from Ashe and Buncombe counties. Similarly, in adults, a prevalence of 0.05 was recorded in Buncombe County and 0.02 in Allegheny County. With *A. phagocytophilum*, low nymph infection prevalence (0.01–0.03) was registered in Alleghany, Ashe, Buncombe, Mitchell, and Watauga (Tables 1, 2). With adults, an alarming high prevalence of 0.2 was registered in Mitchell County, although the sample size was small (n = 5) (Table 2). Other counties with adult ticks positive for *A. phagocytophilum* included Ashe (0.02) and Buncombe (0.05) counties (Table 2). A few cases of co-infection of *I. scapularis* with *B. burgdorferi* and *A. phagocytophilum* were recorded in Alleghany (n = 2), Ashe (n = 1), Watauga (n = 2), and Buncombe (n = 1) counties (Table 1, 2).

## Discussion

LD distribution in NC appears to be changing rapidly. Whereas in the past, LD human cases and *I. scapuilaris* Bb infections appeared sporadic and limited to the Coastal Plain and Piedmont provinces of the state [8,9,26,27], it has now expanded into the Blue Ridge Mountains of western NC. A spatiotemporal analysis of LD cases in Virginia and North Carolina by Lantos et al. [6] indicated a gradual northeast-to-southwest trend in human case distribution along the Blue Ridge Mountains. The study also predicted an imminent increase in LD cases in the northwestern region of NC. Indeed, Mokashi et al. [7] reported that the Mountains region has experienced the highest degree of increase in LD case numbers, with a relatively small or no change in the Coastal Plain or the Piedmont provinces. However, the driving force underlying this change was not clear. That was the goal of this study. In our study, we hypothesized that this expanding range in geographic distribution of human LD cases is driven by a change in the distribution of *I. scapularis* (and the pathogens they carry) invading northwestern NC from southwestern Virginia along the Blue Ridge Mountains. Hence, we predicted to: (1) find higher density of *I. scapularis* and Bb infection prevalence in the Blue Ridge Mountains province compared with the Piedmont and the Coastal regions, and (2) within the Blue Ridge Mountains, we expected to find a north-to-south decreasing gradient in *I. scapularis* tick density and Bb infection prevalence.

### Comparison of *I. scapularis* density and Bb infection among physiographic provinces

The results of our statewide survey are, generally, consistent with our first prediction. In terms of tick distribution and abundance, the Blue Ridge Mountains of NC is now characterized by high density and contiguous distribution of *I. scapularis* whereas in the Coastal Plain and the Piedmont, *I. scapularis* density is lower and distribution is more sporadic. Specifically, this pattern is consistent for nymph but is less clear for adults but that might be related to our low coverage of the coastal region. Indeed, this issue of under-sampling of the coastal region (which was the result of limited manpower and our focus on regions where most change in human LD was reported from Mokashi et al. [7]) is a limitation of our study, and further research on *I. scapularis* distribution, abundance, ecology, and infection patterns in that part of the state is required. It is accepted, though, that *I. scapularis* ticks in this region (i.e., coast and probably the eastern Piedmont) belong to the ‘southern clade’ [12,28], which are characterized by different questing behavior compared with ‘northern clade’ ticks [12,29], with ‘southern clade’ ticks questing lower (within or close to the leaf litter area) and mostly parasitizing lizards [9,10,30] whereas ‘northern clade’ ticks tend to quest higher and therefore are more likely to attach to above-leaf litter mammals including humans thereby posing a greater infection risk to humans [12,30]. *Ixodes scapularis* ticks in this study were not genetically analyzed, so at this point, we cannot ascertain if ticks in the Blue Ridge Mountains province belong to the “northern clade” and those from the coast belong to the “southern clade”. This would be our next step. Particularly interesting would be the evaluation of potential hybrids in the Piedmont region where, potentially “southern” and “northern” clade tick distributions may converge, with unknown potential epidemiological consequences.

Our analysis of *I. scapularis* presence status indicated that between 1996–2015 some change in *I. scapularis* has occurred, mostly in the Coastal Plain and Piedmont provinces. In those earlier studies, only one or two counties in the Blue Ridge Mountains province were found to have *I. scapularis* ticks [26,27]. In contrast, our study indicated a dramatic shift in tick distribution in the Blue Ridge Mountains province, with 13 counties identified as ‘established’ (10) or ‘reported’ (3). In the Piedmont, we observed that most change has occurred within the western part of the Piedmont, suggesting that a possible spill-over of *I. scapularis* ticks from the Blue Ridge Mountains into the Piedmont might be occurring. Indeed, in a recent study based on ticks collected from harvested white-tailed deer in the western Piedmont of NC, a significant north-to-south decreasing gradient in *I. scapularis* tick infestation rate and Bb infection prevalence was reported [31]. Finally, due to our limited sampling in that region, our study could not shed any new light on any change in tick presence status in the Coastal Plain province.

In terms of Bb infection prevalence patterns, results are also consistent with our prediction, with both nymphs and adults exhibiting highest infection prevalence in the Blue Ridge Mountains compared with the Piedmont and the Coastal Plain provinces. Specifically, with nymphs, Bb infection prevalence in the Piedmont was 3% with all infected nymphs coming from the western county of Surry, bordering the Mountains region and Virginia. No infected nymphs were found in the Coastal region. With adults, no Bb infections were found in ticks collected in the Piedmont (but note n = 13) and only 4% in those collected in the coastal region (all coming from Johnston County). The latter is consistent with Mokashi et al. [7] who reported 0–2 LD cases/100,000 people from that county in 2019–2020.

Our study did not test for fine-scale temporal changes in tick density during the study period (2018–2023) because, due to logistic manpower limitations, not all counties could be surveyed at the same time at all years. We did account for the effect of year and sampling effort in our statistical analyses. However, for counties that were sampled consistently during the study period (like Ashe, Alleghany, and Watauga), no consistent temporal trend was observed.

### *Ixodes scapularis* density and Bb infection gradients within the Blue Ridge Mountains province

As predicted the by our ‘Virginia invasion hypothesis’, a clear northeast-to-southwest decreasing gradient in *I. scapularis* density and *Bb* infection prevalence was observed. For both *I. scapularis* nymphs and adults, counties with highest density and Bb infection prevalence occurred close to the border with Virginia while in the more southwestern counties, *I. scapularis* tick density and *B. burgdorferi* infection prevalence were lower or absent altogether. The southwestern most county reporting the presence of *I. scapularis* in our study was Haywood County. However, a more recent 2023/2024 survey (not included in this report) documented nine *I. scapularis* specimens (nymphs and adults) in the far southwestern county of Swain (Seagle, pers. comm.). In addition to the high infection prevalence reported near the Virginia-NC border, another, disjointed, region of high infection prevalence occurred in and around Buncombe County, which is the location of Asheville, the largest urban hub in the Blue Ridge Mountains. This finding is consistent with the LD outbreak reported by Barbarin et al. [18] and suggests a potential anthropogenic effect facilitating *I. scapularis* tick establishment and pathogen transmission. Yet, the data presented here for Buncombe County was based on the entomologic survey conducted by Barbarin et al. [18] following this outbreak. Hence, this data may overrepresent the entomological risk level for this county and therefore should be treated with caution and more studies in other parts of Buncombe County are warranted.

Another observation worth noting is that the geographic range of infected ticks extends less far south compared to the broader range of uninfected *I. scapularis* ticks. This distribution pattern aligns with the “tick-first hypothesis,” which was proposed to explain the northward expansion of LD into Michigan from Indiana [32]. In this hypothesis, new uninfected *I. scapularis* populations become established first due to gradual or long-distance dispersal of adult ticks by typically white-tailed deer. This is followed by slower secondary invasion mediated by infected mammalian or avian hosts [32]. Clearly, this idea warrants further research to evaluate the relative support of this versus other alternative hypotheses.

### Infection patterns of *I. scapularis* in NC with other tick-borne pathogens

In addition to infections with *B. burgdorferi* we also detected *I. scapularis* ticks infected with *B. miyamotoi* (causal agent of hard tick relapsing fever) and *A. phagocytophilum* (agent of human granulocytic anaplasmosis)*.* No infections with *Babesia microti or B. mayonii* were detected. To the best of our knowledge, our study is the first to report hard ticks infected with *B. miyamotoi and A. phagocytophilum* in the state although *Anaplasma* spp. and *B. burdorferi* infections were recorded in dogs, racoons, and black bears in eastern NC in the past [33,34,35,36]. In our study, these pathogens were found only in the Blue Ridge Mountains province with infection prevalence typically low, but with some counties (e.g., Mitchell County) reporting prevalence as high as 20% for *A. phagocytophilum*. *Babesia microti* was detected in the past in the in neighboring states of Virginia and West Virginia, with autochthonous human cases reported between 2009 and 2024 [37]. *Borrelia miyamotoi* infections were found in *I. scapularis* collected in Virginia from human-biting ticks (1.2%) [38]. Our study did not screen for tickborne viruses. Indeed, Powassan virus, causing a rare but serious tick-borne illness, has been detected in blacklegged ticks in North Carolina and neighboring states, highlighting the need for continued surveillance [39]. Our findings suggest that the expanding range and abundance of *I. scapularis* has the potential to increase human risk to other tick-borne pathogens in this part of the state.

## Conclusions

Overall, the results of this study clearly indicate that the Blue Ridge Mountains province is now the most endemic Lyme disease (LD) region in the state, characterized by the highest human case incidence [7], the greatest *I. scapularis* tick densities, and the highest Bb infection prevalence in ticks. Tick densities and infection rates in this area, as observed in this study, are comparable to those reported in some hyperendemic LD regions of the Northeast, Mid-Atlantic, and Upper Midwest [40,41]. For example, nymph densities (3.49 nymphs/100 m) and infection levels (39%) observed in our hot-spot counties of Ashe and Alleghany are comparable to those reported in Wisconsin (1.58 nymphs/100 m and 40–45% prevalence) and Connecticut (3.22 nymphs/100 m and 45–48% prevalence) [40–42]. Our findings are consistent with the view that this shift in tick distribution is recent and linked to the ongoing southwesterly expansion of northern clade ticks along the Blue Ridge Mountains. As time goes by, it is possible that the *I. scapularis* population in the Blue Ridge Mountains will continue to increase and spread further southwest along the Appalachian Mountain range and possibly spill-over into the western Piedmont. Therefore, continued monitoring of *I. scapularis*, and screening for their associated pathogens, together with further ecological research on the underlying enzootic (e.g., study of the wildlife reservoir community) and environmental variables that affect transmission in NC is needed. This should be overlain with continuous monitoring of LD and other *I. scapularis*-borne disease cases in humans, and companion animals. This approach is already taking place in NC, depicting the adoption of the One Health approach for managing LD spread in the state [43,44]. Understanding the distribution and abundance of *I. scapularis* ticks and the pathogens they carry together with understanding the underlying ecological and sociological drivers of these diseases is essential for developing an evidence-based approach aimed at increased awareness of *I. scapularis*-borne pathogens (with a focus on LD) among healthcare professionals and the public at large as well to minimize delayed diagnosis and reduce exposure to *I. scapularis* tick bites.

## Supporting information

S1 Table Summary table of the *I. scapularis* cumulative presence status in NC. (DOCX)

S2 Fig Sampling sites map. (TIF)

S3 Fig Land-use cover map of surveyed area. (TIF)

S4 File Statistical tables. (DOCX)

S5 File Statistics for generating figures 1 and 2. (DOCX)
