# Supplementary material for: Expanding range of Ixodes scapularis Say (Acari: Ixodidae) and Borrelia burgdorferi infection in North Carolina counties, 2018–2023
Source: PLoS One. 2025 Aug 13;20(8):e0329511. doi: 10.1371/journal.pone.0329511 (PMC12349693; doi:10.1371/journal.pone.0329511)
Supplement: S1 Table — (DOCX) [file pone.0329511.s001.docx]

**S1.** Summary table of the *I. scapularis* (cumulative) presence status in the different counties surveyed and the amount of effort, number of sites, and the years involved.

| **Region** | **County** | **Status** | **Effort (m^2^)** | **Sites** | **Year** |
| --- | --- | --- | --- | --- | --- |
| **Blue Ridge Mountains** | Alleghany | Established | 21200 | 2 | 2018, 2019, 2020, 2021 |
|  | Ashe | Established | 26100 | 2 | 2018, 2019, 2020, 2021 |
|  | Avery | Reported | 8400 | 5 | 2018, 2019, 2020 |
|  | Buncombe | Established | 42077 | 20 | 2019, 2020, 2021 |
|  | Burke | Not detected | 9600 | 2 | 2020, 2021, 2022, 2023 |
|  | Caldwell | Established | 4800 | 2 | 2020 |
|  | Haywood | Established | 4800 | 2 | 2019, 2020 |
|  | Henderson | Reported | 7200 | 5 | 2022, 2023 |
|  | Jackson | Not detected | 6450 | 5 | 2021, 2022 |
|  | Macon | Not detected | 8200 | 6 | 2021, 2022 |
|  | Madison | Established | 2400 | 2 | 2019 |
|  | McDowell | Reported | 6250 | 5 | 2020, 2021 |
|  | Mitchell | Established | 4120 | 4 | 2019 |
|  | Polk | Not detected | 6600 | 4 | 2022, 2023 |
|  | Rutherford | Reported | 6600 | 4 | 2022, 2023 |
|  | Transylvania | Not detected | 3700 | 3 | 2023 |
|  | Watauga | Established | 25240 | 19 | 2018, 2019, 2020, 2021, 2023 |
|  | Wilkes | Established | 10000 | 3 | 2018, 2019, 2021 |
|  | Yancey | Established | 2400 | 2 | 2019 |
| **Piedmont** | Alamance | Not detected | 4800 | 2 | 2020, 2021 |
|  | Mecklenburg | Reported | 46360 | 27 | 2019, 2020, 2021, 2022, 2023 |
|  | Montgomery | Reported | 14000 | 2 | 2021, 2022 |
|  | Union | Not detected | 6000 | 4 | 2021, 2022 |
|  | Alexander | Reported | 4800 | 2 | 2020 |
|  | Anson | Reported | 3600 | 1 | 2022, 2023 |
|  | Catawba | Not detected | 6600 | 4 | 2020, 2021 |
|  | Chatham | Established | 112000 | 5 | 2020, 2021, 2022 |
|  | Davie | Not detected | 4800 | 2 | 2020, 2021 |
|  | Durham | Not detected | 50000 | 2 | 2020, 2021, 2022 |
|  | Forsyth | Not detected | 12400 | 2 | 2020, 2021, 2022 |
|  | Guilford | Established | 6000 | 2 | 2021, 2022, 2023 |
|  | Iredell | Not detected | 8400 | 2 | 2020, 2021 |
|  | Orange | Reported | 50000 | 2 | 2020, 2021, 2022 |
|  | Stanly | Not detected | 3600 | 1 | 2022 |
|  | Surry | Established | 5900 | 2 | 2018, 2019 |
|  | Vance | Established | 40200 | 2 | 2020, 2021, 2022, 2023 |
|  | Wake | Reported | 8500 | 2 | 2019, 2021 |
|  | Yadkin | Reported | 4000 | 1 | 2018 |
| **Coastal Plain** | Camden | Established | 2400 | 1 | 2023 |
|  | Craven | Established | 2400 | 2 | 2022, 2023 |
|  | Johnston | Reported | 3500 | 2 | 2020 |
|  | Onslow | Reported | 4000 | 2 | 2019 |
