# Supplementary figures and images for: Expanding range of Ixodes scapularis Say (Acari: Ixodidae) and Borrelia burgdorferi infection in North Carolina counties, 2018–2023
Source: PLoS One. 2025 Aug 13;20(8):e0329511. doi: 10.1371/journal.pone.0329511 (PMC12349693; doi:10.1371/journal.pone.0329511)

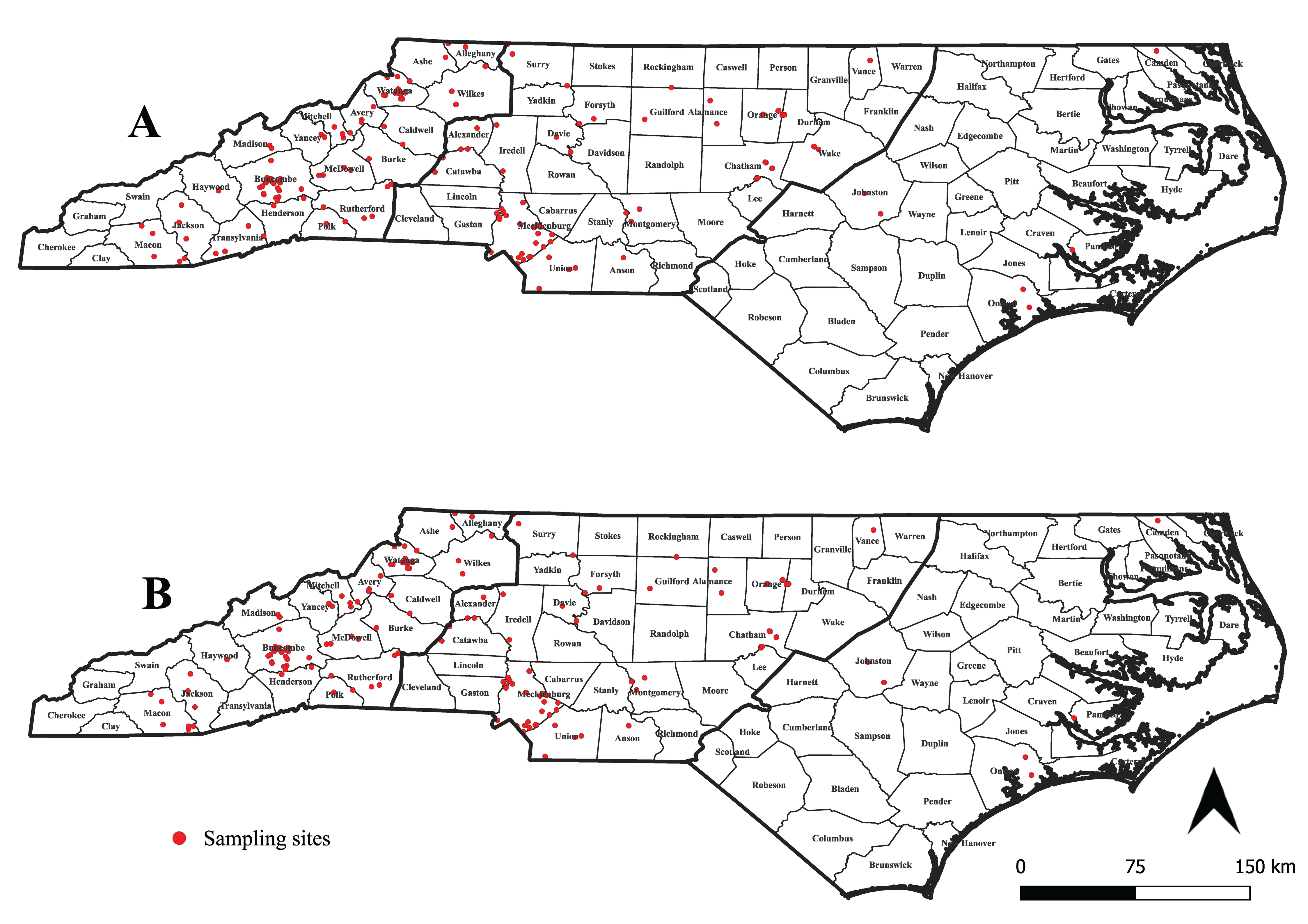

Supplement: S2 Fig — (TIF) [file pone.0329511.s002.tif]

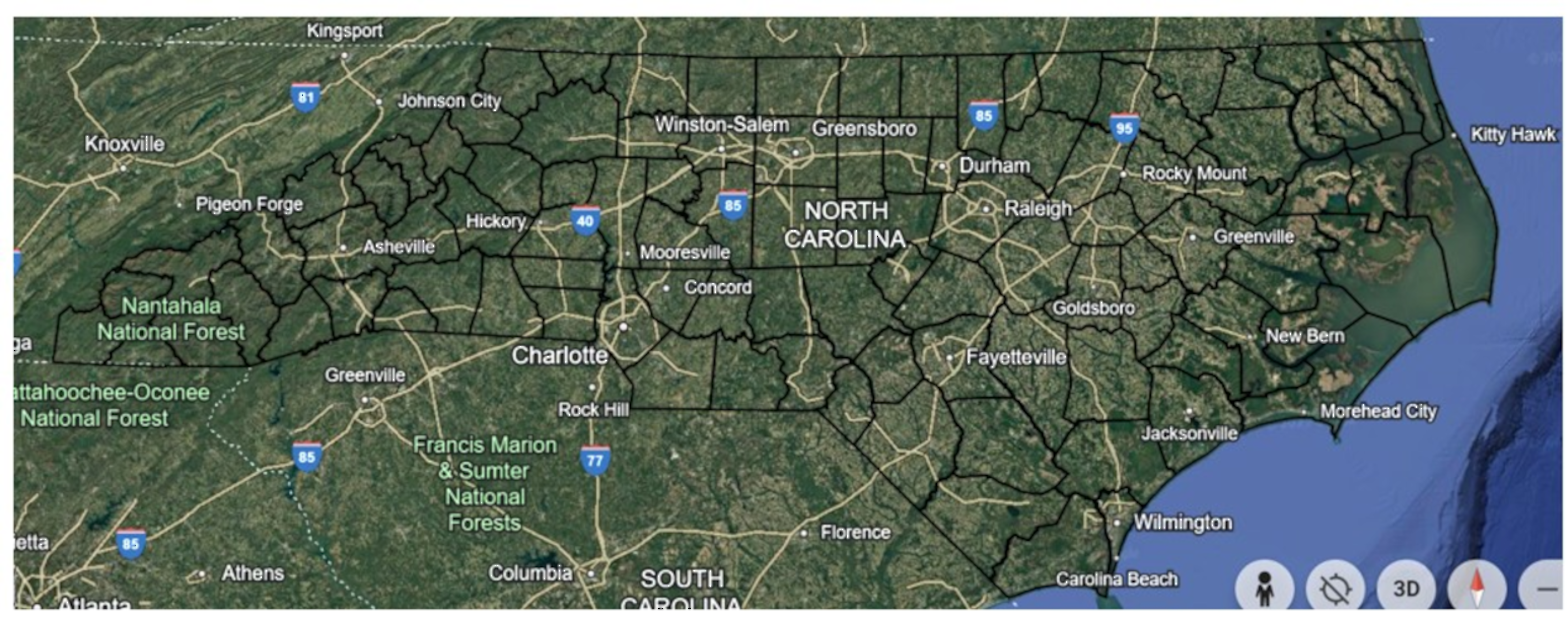

Supplement: S3 Fig — (TIF) [file pone.0329511.s003.tif]
