# Supplementary material for: Expanding range of Ixodes scapularis Say (Acari: Ixodidae) and Borrelia burgdorferi infection in North Carolina counties, 2018–2023
Source: PLoS One. 2025 Aug 13;20(8):e0329511. doi: 10.1371/journal.pone.0329511 (PMC12349693; doi:10.1371/journal.pone.0329511)
Supplement: S4 File — (DOCX) [file pone.0329511.s004.docx]

| **Fixed effects** | **Estimates** | **Std. error** | **z-value** | **p-value** |
| --- | --- | --- | --- | --- |
| Intercept | -4.52 | 0.87 | -5.19 | 2.07E-07 |
| Latitude_scaled | 1.29 | 0.55 | 2.36 | 0.019 |
| **Random effects** |  |  |  |  |
| County | 3.76 | 1.94 | - | - |
| Location | 2.36 | 1.54 | - | - |
| Year | 0.25 | 0.50 | - | - |
| **Model fit statistics** |  |  |  |  |
| AIC | 390.80 | - | - | - |
| BIC | 408.50 | - | - | - |
| Log-Likelihood | -189.40 | - | - | - |
| Deviance | 378.80 | - | - | - |
| Residual df | 136.00 | - | - | - |
| **Dispersion parameter** |  |  |  |  |
| θ (nbinom2) | 1.51 | - | - | - |
| **R^2^ value** |  |  |  |  |
| Conditional R^2^ | 0.59 | - | - | - |

**S4. Statistical analysis tables**

**Table 1.** Summary of the best negative binomial generalized linear mixed model of *Ixodes scapularis* nymph density against latitude (scaled), for the Blue Ridge Mountains region, with substantive random intercepts and number of transects as offset.

**Table 2.** Summary of the best negative binomial generalized linear mixed model of *Ixodes scapularis* adult density against latitude (scaled), for the Blue Ridge Mountains region, with substantive random intercept and number of transects as offset.

| **Fixed effects** | **Estimates** | **Std. error** | **z-value** | **p-value** |
| --- | --- | --- | --- | --- |
| Intercept | -5.10 | 0.80 | -6.41 | 1.50E-10 |
| Latitude_scaled | 1.12 | 0.45 | 2.48 | 0.013 |
| **Random effects** |  |  |  |  |
| Year | 1.40 | 1.18 | - | - |
| **Model fit statistics** |  |  |  |  |
| AIC | 72.50 | - | - | - |
| BIC | 81.20 | - | - | - |
| Log-Likelihood | -32.20 | - | - | - |
| Deviance | 64.50 | - | - | - |
| Residual df | 61.00 | - | - | - |
| **Dispersion parameter** |  |  |  |  |
| θ (nbinom1) | 9.23E-09 | - | - | - |
| **R^2^ value** |  |  |  |  |
| Conditional R^2^ | 0.38 | - | - | - |

**Table 3.** Summary of the best binomial generalized linear mixed model of *Borrelia burgdorferi* infection probability in *Ixodes scapularis* nymphs against latitude (scaled), for the Blue Ridge Mountains region, with the substantive random intercepts.

| **Fixed effects** | **Estimates** | **Std. error** | **z-value** | **p-value** |
| --- | --- | --- | --- | --- |
| Intercept | -3.01 | 0.66 | -4.58 | 4.72E-06 |
| Latitude_scaled | 1.00 | 0.46 | 2.18 | 0.029 |
| **Random effects** |  |  |  |  |
| County:Location | 1.36 | 1.17 | - | - |
| County | 1.19 | 1.09 | - | - |
| Year | 0.29 | 0.54 | - | - |
| **Model fit statistics** |  |  |  |  |
| AIC | 245.20 | - | - | - |
| BIC | 255.70 | - | - | - |
| Log-Likelihood | -117.60 | - | - | - |
| Deviance | 235.20 | - | - | - |
| Residual df | 56.00 | - | - | - |
| **R^2^ value** |  |  |  |  |
| Conditional R^2^ | 0.54 | - | - | - |

**Table 4.** Summary of the best binomial generalized linear mixed model of *Borrelia burgdorferi* infection probability in *Ixodes scapularis* adults against latitude (scaled), for the Blue Ridge Mountains region, with substantive random intercepts.

| **Fixed effects** | **Estimates** | **Std. error** | **z-value** | **p-value** |
| --- | --- | --- | --- | --- |
| Intercept | -1.25 | 0.83 | -1.5 | 1.33E-01 |
| Latitude_scaled | 1.07 | 0.44 | 2.45 | 0.014 |
| **Random effects** |  |  |  |  |
| County | 0.95 | 0.97 | - | - |
| Year | 0.51 | 0.71 | - | - |
| **Model fit statistics** |  |  |  |  |
| AIC | 93.10 | - | - | - |
| BIC | 99.70 | - | - | - |
| Log-Likelihood | -42.60 | - | - | - |
| Deviance | 85.10 | - | - | - |
| Residual df | 34.00 | - | - | - |
| **R^2^ value** |  |  |  |  |
| Conditional R^2^ | 0.44 | - | - | - |

**Table 5.** Summary of the best negative binomial generalized linear mixed model of *Ixodes scapularis* nymph density by physiographic region using the Blue Ridge Mountains region as reference, with substantive random intercepts and number of transects as offset.

| **Fixed effects** | **Estimates** | **Std. error** | **z-value** | **p-value** |
| --- | --- | --- | --- | --- |
| Intercept | -4.09 | 0.65 | -6.33 | 2.43E-10 |
| Coastal | -2.95 | 2.18 | -1.35 | 1.77E-01 |
| Piedmont | -3.00 | 0.93 | -3.23 | 0.0012 |
| **Random effects** |  |  |  |  |
| County:Location | 1.68 | 1.30 | - | - |
| County | 3.17 | 1.78 | - | - |
| Year | 0.34 | 0.59 | - | - |
| **Model fit statistics** |  |  |  |  |
| AIC | 482.40 | - | - | - |
| BIC | 508.10 | - | - | - |
| Log-Likelihood | -234.20 | - | - | - |
| Deviance | 468.40 | - | - | - |
| Residual df | 282.00 | - | - | - |
| **Dispersion parameter** |  |  |  |  |
| θ (nbinom2) | 2.97 | - | - | - |
| **R^2^ value** |  |  |  |  |
| Conditional R^2^ | 0.56 | - | - | - |

**Table 6.** Summary of the best negative binomial generalized linear mixed model of *Ixodes scapularis* adult density by physiographic region using the Blue Ridge Mountains region as reference, with substantive random intercepts and number of transects as offset.

| **Fixed effects** | **Estimates** | **Std. error** | **z-value** | **p-value** |
| --- | --- | --- | --- | --- |
| Intercept | -3.88 | 0.38 | -10.2 | < 2E-16 |
| Coastal | 0.21 | 0.66 | 0.31 | 7.57E-01 |
| Piedmont | -3.47 | 1.07 | -3.25 | 0.0012 |
| **Random effects** |  |  |  |  |
| Year | 0.32 | 0.57 | - | - |
| **Model fit statistics** |  |  |  |  |
| AIC | 114.60 | - | - | - |
| BIC | 130.50 | - | - | - |
| Log-Likelihood | -52.30 | - | - | - |
| Deviance | 104.60 | - | - | - |
| Residual df | 174.00 | - | - | - |
| **Dispersion parameter** |  |  |  |  |
| θ (nbinom2) | 6.20E+07 | - | - | - |
| **R^2^ value** |  |  |  |  |
| Conditional R^2^ | 0.39 | - | - | - |

**Table 7.** Summary of the simple logistic model used to model the effect of physiographic region on *Ixodes scapularis* nymph infection probability using Blue Ridge Mountains as reference. Note that the sample size for the coastal plain was only four.

| **Parameters** | **Estimates** | **Std. error** | **z-value** | | **p-value** |
| --- | --- | --- | --- | --- | --- |
| Intercept | -1.31 | 0.08 | -16.67 | < 2E-16 | |
| Coastal | -14.85 | 1130.53 | -0.013 | 7.57E-01 | |
| Piedmont | -0.82 | 0.32 | -2.60 | 0.0094 | |
| **Model fit statistics** |  |  |  |  | |
| Null deviance | 275.75 | - | - | - | |
| Residual deviance | 266.32 | - | - | - | |
| AIC | 351.88 | - | - | - | |
| **Dispersion parameter** |  |  |  |  | |
| ϥ (binomial) | 1.00E+00 | - | - | - | |
| **R^2^ value** |  |  |  |  | |
| Conditional R^2^ | 0.04 | - | - | - | |

**Table 8.** Summary of the simple logistic model used to model the effect of physiographic region on *Ixodes scapularis* adult infection probability using Coastal Plain region as model reference.

| **Fixed effects** | **Estimates** | **Std. error** | **z-value** | **p-value** |
| --- | --- | --- | --- | --- |
| Intercept | -3.09 | 0.72 | -4.28 | 1.91E-05 |
| Blue Ridge | 3.10 | 0.74 | -4.2 | 2.67E-05 |
| Piedmont | -14.62 | 1229.14 | -0.01 | 0.991 |
| **Model fit statistics** |  |  |  |  |
| Null deviance | 154.34 | - | - | - |
| Residual deviance | 102.54 | - | - | - |
| AIC | 138.47 | - | - | - |
| **Dispersion parameter** |  |  |  |  |
| ϥ (binomial) | 1.00E+00 | - | - | - |
| **R^2^ value** |  |  |  |  |
| Conditional R^2^ | 0.33 | - | - | - |

**Table 9:** Summary of the best GLMM model for the effect of year on *I. scapularis* nymph density for consecutive years (2018-2021) for the counties of Ashe, Alleghany, and Watauga.

| **Fixed effects** | **Estimates** | **Std. error** | **z-value** | **p-value** |
| --- | --- | --- | --- | --- |
| Intercept | -4.00 | -0.43 | -6.33 | 0.667 |
| Year | -0.21 | 0.51 | -0.40 | 0.686 |
| **Random effects** |  |  |  |  |
| Location | 3.26 | 1.81 | - | - |
| **Model fit statistics** |  |  |  |  |
| AIC | 191.20 | - | - | - |
| BIC | 197.40 | - | - | - |
| Log-Likelihood | -91.60 | - | - | - |
| Deviance | 183.20 | - | - | - |
| Residual df | 31.00 | - | - | - |
| **Dispersion parameter** |  |  |  |  |
| θ (nbinom2) | 1.70 | - | - | - |
| **R^2^ value** |  |  |  |  |
| Conditional R^2^ | 0.72 | - | - | - |

**Table 10:** Summary of the best GLMM model for the effect of year on *I. scapularis* adult density for consecutive years (2018-2021) for the counties of Ashe, Alleghany, and Watauga.

| **Fixed effects** | **Estimates** | **Std. error** | **z-value** | **p-value** |
| --- | --- | --- | --- | --- |
| Intercept | -3.73 | 0.99 | -3.77 | 1.63E-04 |
| Year | -0.47 | 0.99 | -0.47 | 0.635 |
| **Random effects** |  |  |  |  |
| Location | 3.26 | 1.81 | - | - |
| **Model fit statistics** |  |  |  |  |
| AIC | 191.20 | - | - | - |
| BIC | 197.40 | - | - | - |
| Log-Likelihood | -91.60 | - | - | - |
| Deviance | 183.20 | - | - | - |
| Residual df | 31.00 | - | - | - |
| **Dispersion parameter** |  |  |  |  |
| θ (nbinom2) | 1.70 | - | - | - |
| **R^2^ value** |  |  |  |  |
| Conditional R^2^ | 0.72 | - | - | - |
