# Supplementary material for: Expanding range of Ixodes scapularis Say (Acari: Ixodidae) and Borrelia burgdorferi infection in North Carolina counties, 2018–2023
Source: PLoS One. 2025 Aug 13;20(8):e0329511. doi: 10.1371/journal.pone.0329511 (PMC12349693; doi:10.1371/journal.pone.0329511)
Supplement: S5 File — (DOCX) [file pone.0329511.s005.docx]

**Appendix A (Fig. 1a):** Statistics used to determine the density of *Ixodes scapularis* nymphs: total number of transects (a), total number of nymphs collected (b), nymph density (b/a), and the standard error (standard deviation/√(b)) for each of the three physiographic regions of North Carolina. The Dunn test using the ‘bonferroni’ method was used to determine significance. Two regions with different variables attached to their densities indicate significant difference and vice versa.

| **Region** | **# transects** | **Nymphs** | **Density** | **Std. dev.** | **Std. error** |
| --- | --- | --- | --- | --- | --- |
| Blue Ridge | 1504 | 1740 | 1.16a | 2.83 | 0.2360 |
| Piedmont | 1967 | 102 | 0.052b | 0.25 | 0.0213 |
| Coastal | 91 | 4 | 0.044b | 0.09 | 0.0354 |

**Appendix B (Fig. 2b):** Statistics used to determine the density of *Ixodes scapularis* adults: total number of transects (a), total number of adults collected (b), adult density (b/a), and the standard error (standard deviation/√(b)) for each of the three physiographic regions of North Carolina. The Dunn test using the ‘bonferroni’ method was used to determine significance. Two regions with different variables attached to their densities indicate significant difference and vice versa.

| **Region** | **# transects** | **Adults** | **Density** | **Std. dev.** | **Std. error** |
| --- | --- | --- | --- | --- | --- |
| Blue Ridge | 728 | 299 | 0.411a | 0.453 | 0.0562 |
| Piedmont | 1958 | 25 | 0.0128b | 0.025 | 0.0025 |
| Coastal | 81 | 46 | 0.568c | 0.403 | 0.1340 |

**Appendix C (Fig. 1d):** Statistics showing the process for the derivation of the *Borrelia burgdorferi* prevalence in *Ixodes scapularis* nymphs for the three different physiographic regions: total number of tested nymphs (X), total number of *B. burgdorferi*-infected nymphs (Y), prevalence (Y/X = Z), and standard error [√ (Z * (1 – Z)/X]. Two regions with different variables attached to their densities indicate significance and vice versa.

| **Region** | **# tested** | **# infected** | **Prevalence** | **Std. error** |
| --- | --- | --- | --- | --- |
| Blue Ridge | 175 | 88 | 0.503a | 0.0378 |
| Piedmont | - | - | - | - |
| Coastal | 46 | 2 | 0.044b | 0.0301 |

**Appendix D (Fig. 2d):** Statistics showing the process for the derivation of the *Borrelia burgdorferi* prevalence in *Ixodes scapularis* adults for the three different physiographic regions: total number of tested adults (X), total number of *B. burgdorferi*-infected adults (Y), prevalence (Y/X = Z), and standard error [√ (Z * (1 – Z)/X]. Two regions with different variables attached to their densities indicate significance and vice versa.

| **Region** | **# tested** | **# infected** | **Prevalence** | **Std. error** |
| --- | --- | --- | --- | --- |
| Blue Ridge | 966 | 205 | 0.212a | 0.0132 |
| Piedmont | 113 | 12 | 0.106b | 0.0290 |
| Coastal | - | - | - | - |
